# Update on Poly ADP-Ribose Polymerase Inhibitors in Ovarian Cancer With Non-BRCA Mutations

**DOI:** 10.3389/fphar.2021.743073

**Published:** 2021-11-29

**Authors:** Qin Xu, Zhengyu Li

**Affiliations:** Department of Obstetrics and Gynecology, Key Laboratory of Birth Defects and Related Diseases of Women and Children, Ministry of Education, West China Second University Hospital, Sichuan University, Chengdu, China

**Keywords:** poly ADP-ribose polymerase inhibitors, homologous recombination deficiency, ovarian cancer, combination therapy, HRD status detection

## Abstract

Poly ADP-ribose polymerase inhibitor (PARPi) has become an important maintenance therapy for ovarian cancer after surgery and cytotoxic chemotherapy, which has changed the disease management model of ovarian cancer, greatly decreased the risk of recurrence, and made the prognosis of ovarian cancer better to certain extent. The three PARPis currently approved by the United States Food and Drug Administration (FDA) and the European Medicines Agency (EMA) for the treatment of ovarian cancer are Olaparib, Niraparib and Rucaparib. With the incremental results from new clinical trials, the applicable population of PARPi for ovarian cancer have expanded to population with non-BRCA mutations. Although BRCA mutated population are still the main beneficiaries of PARPi, recent clinical trials indicated PARPis’ therapeutic potential in non-BRCA mutated population, especially in homologous recombination repair deficiency (HRD) positive population. However, lack of unified HRD status detection method poses a challenge for the accurate selection of PARPi beneficiaries. The reversal of homologous recombination (HR) function during the treatment will not only cause resistance to PARPis, but also reduce the accuracy of the current method to determine HRD status. Therefore, the development of reliable HRD status detection methods to determine the beneficiary population, as well as rational combination treatment are warranted. This review mainly summarizes the latest clinical trial results and combination treatment of PARPis in ovarian cancer with non-BRCA mutations, and discusses the application prospects, including optimizing combination therapy against drug resistance, developing unified and accurate HRD status detection methods for patient selection and stratification. This review further poses an interesting topic: the efficacy and safety in patients retreated with PARPis after previous PARPi treatment---“PARPi after PARPi”.

## Introduction

Seventy percent of ovarian cancer patients are diagnosed at an advanced stage of disease due to the insidious nature of ovarian cancer and the ineffectiveness of screening tests for early detection (Henderson et al., 2018). At present, surgery and cytotoxic chemotherapy remain the main treatment methods for ovarian cancer. However, the recurrence rate of ovarian cancer is high, and the prognosis is poor. Therefore, on the basis of surgery and cytotoxic chemotherapy, targeted maintenance therapy is needed for some “high-risk” patients with ovarian cancer to improve progression-free survival (PFS) and overall survival (OS) (Oza et al., 2015). Currently, poly ADP-ribose polymerase inhibitors (PARPis) have become a molecularly targeted therapeutic strategy for ovarian cancer (Gadducci and Guerrieri, 2017). Many studies have shown that PARPi can significantly improve the PFS and OS of newly diagnosed and recurrent ovarian cancer patients with breast-related cancer antigen (*BRCA*) mutations, and PARPis have been widely used in *BRCA*-mutated (*BRCA*m) ovarian cancer patients. Homologous recombination repair (HRR) is a key DNA damage repair pathway, and approximately 50% of patients with high-grade serous ovarian cancer (HGSOC) have homologous recombination repair deficiency (HRD) (Sunada et al., 2018). In recent years, studies have found that HRD-positive ovarian cancer patients without *BRCA* mutations can benefit from PARPis, and even HRD-negative ovarian cancer patients have been shown to benefit from PARPis in some studies. Based on increasingly gratifying and reliable research results, the clinical indications for PARPis are constantly expanding beyond the *BRCA*m ovarian cancer population. Studies have found that HRD status is related to the efficacy of PARPis, and non-unified HRD status detection methods pose a certain challenge to accurately select ideal patients to receive PARPis to improve clinical benefits. With the increasing application of PARPis, PARPis drug resistance has gradually emerged. At present, the two main drug-resistance mechanisms of PARPis include the recovery of homologous recombination and the protection of the replication fork (Weigelt et al., 2017; Francica and Rottenberg, 2018). Studies on the combination of PARPis with antiangiogenic drugs, immunoagents, and other biologics to overcome drug resistance are increasing. We reviewed the current research status of PARPis in ovarian cancer patients with non-*BRCA* mutations, the related issues of HRD status detection and PARPis combination therapies and surveyed the application prospect of PARPis in the treatment of ovarian cancer with non-*BRCA* mutations.

## Homologous Recombination Deficiency

The identification and repair of DNA damage are crucial to maintaining normal cell function and genomic stability. Inherited or acquired defects in DNA repair pathways increase the risk of cancer in humans (Hoeijmakers, 2019). DNA damage is typically caused by endogenous and exogenous stimuli: single-strand breaks (SSB) and double-strands breaks (DSB). DSB leads to genomic instability and cell death (Huertas, 2010). DSBs can be repaired through a variety of pathways, and HRR is an error-free way to repair DSBs using homologous DNA templates. When some key homologous recombination genes are damaged or dysregulated, HRD will occur. These genes include BRCA1, BRCA2, BARD1, RAD51B, RAD51C, RAD51D, BRIP1, PALB2, EMSY, CHEK1, CHEK2, ATM, ATR, ATX, BAP1, CDK12, CHEK1, CHEK2, FANCA, FANCC, FANCD2, FANCE, FANCF, PALB2, NBS1, WRN, MRE11A, BLM (Cancer Genome Atlas Research Network, 2011; Walsh et al., 2011; Prakash et al., 2015; Garsed et al., 2018; Son and Hasty, 2019; Janysek et al., 2021). Cells with HRD can only use alternative DNA repair pathways which has lower fidelity than HRR, resulting in a cascade of effects on the genome and increased mutation rates (Nesic et al., 2018). Germline and somatic BRCA mutations account for approximately half of HRD ovarian cancer cases.

## Mechanism of Action of PARP Inhibitors

**FIGURE 1:**
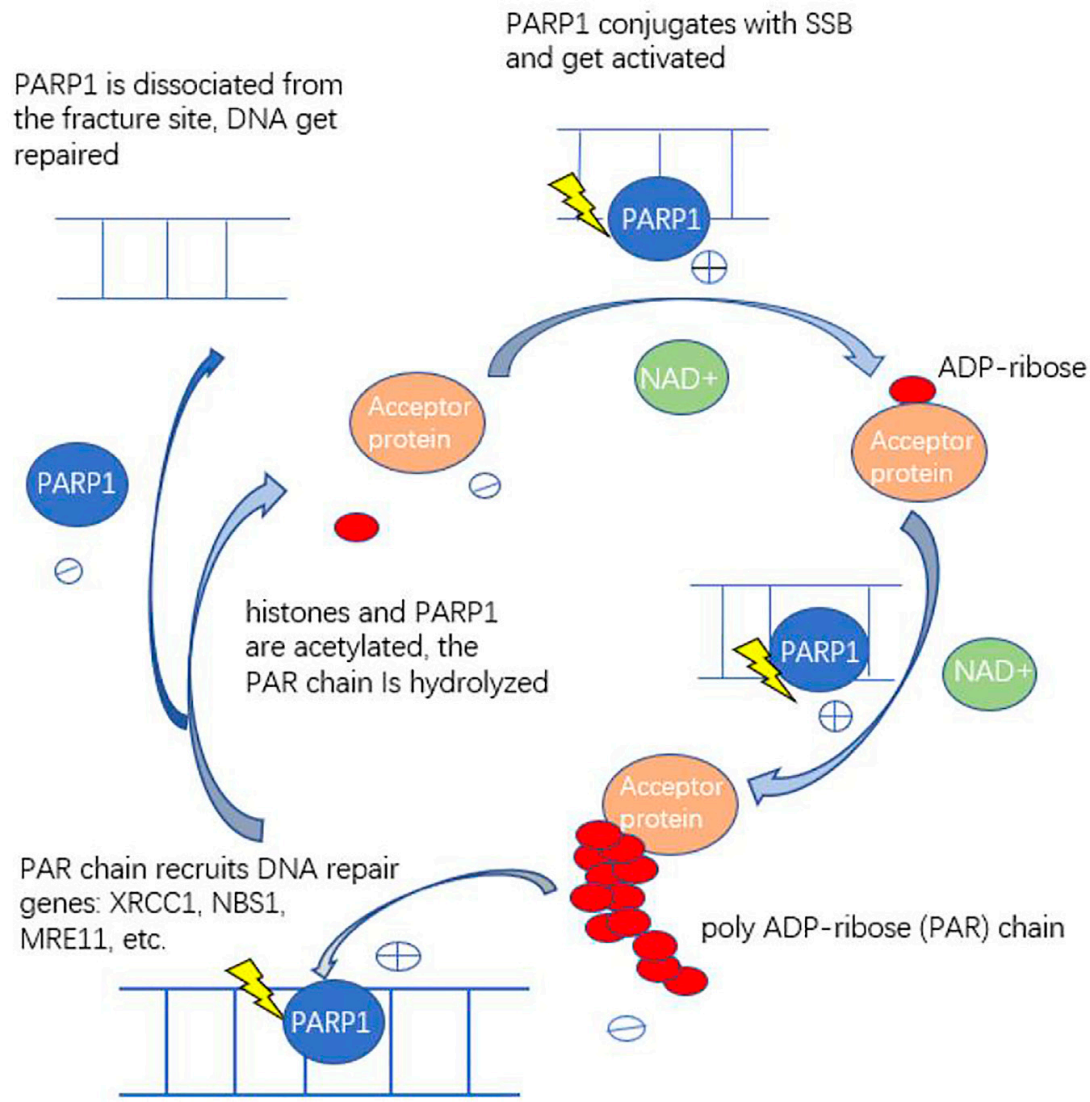
The mechanism of action of PARP1. PARP1 binds to DNA nicks and breaks, which results in activation of catalytic activity causing poly (ADP) ribosylation of PARP1 itself, and of acceptor proteins. The components of DNA repair pathways will be recruited, then DNA get repaired. PARP: poly ADP-ribose polymerase: PAR: poly ADP-ribose: SSB: single-strand breaks.

PARPs are a family of 17 nucleoproteins that share a common catalytic site and use NAD + as a cofactor to transfer the ADP-ribose group to a specific receptor protein. PARP1 is responsible for approximately 90% of the PARylation activity (Langelier et al., 2014). PARP1 conjugates with SSB and then PARP1 gets activated. Activated PARP1 continually cleaves ADP-ribose from NAD+, and then specifically adds ADP-ribose to acceptor proteins, the negatively charged poly ADP-ribose (PAR) chains are produced. PAR chains attached to PARP1 and can recruit DNA repair proteins (XRCC1, NBS1, MRE11, etc.,) to the DNA chain fracture site through electrostatic attraction, and then histones and PARP1 are acetylated. Then, PARP1 is dissociated from the fracture site through the action of electrostatic rejection, allowing other repair pathway proteins to play a role (Figure 1). Poly ADP-ribosyl hydrolase and ADP-ribosyl hydrolase hydrolyze the PAR chain at histones so that histones can rebind to the DNA strand and PARP1 can be activated again, enabling repair of other DNA breaks to start again (Weaver and Yang, 2013).

**FIGURE 2:**
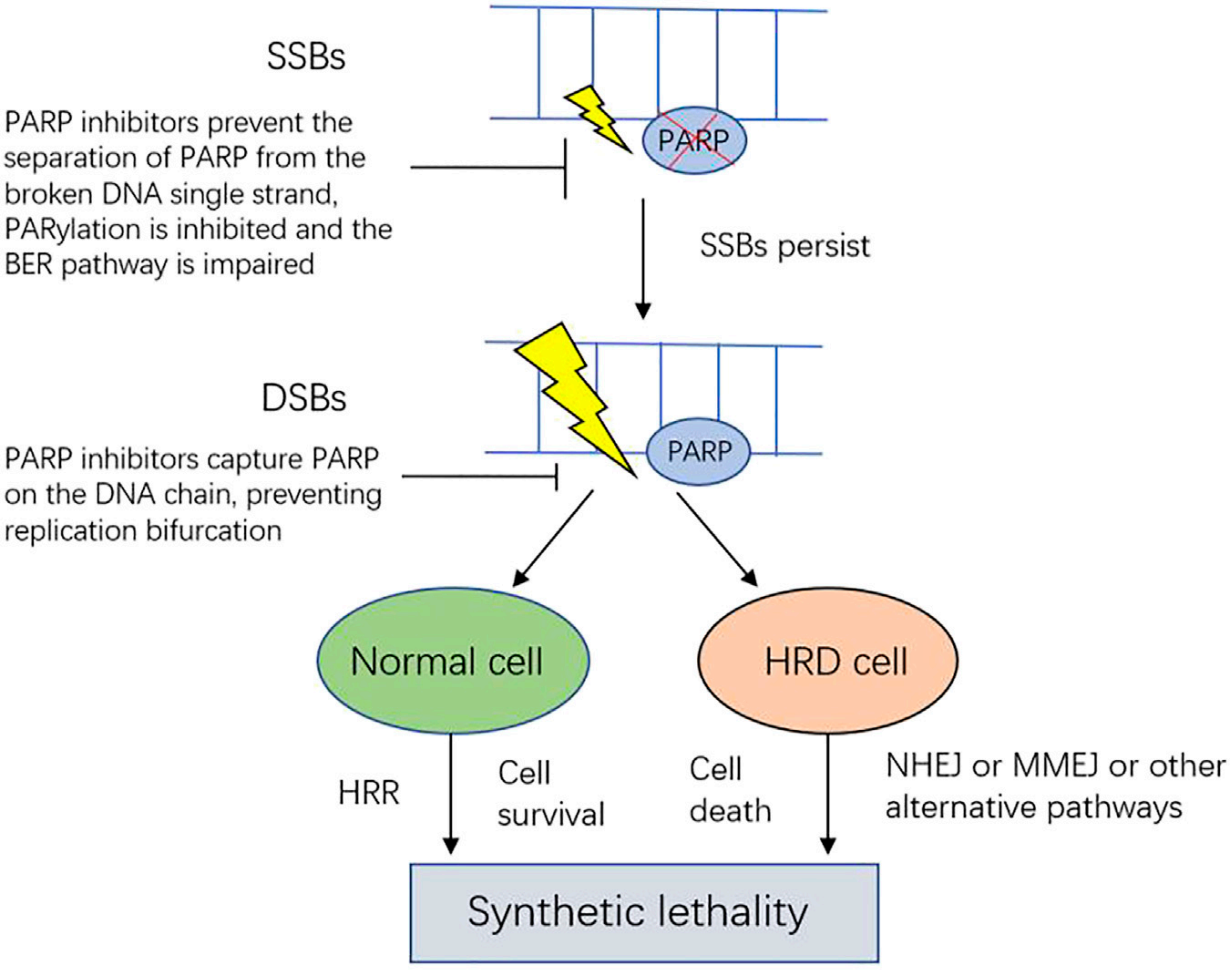
Mechanism of synthetic lethality between HRD and PARP inhibitors. SSBs of DNA are normally efficiently repaired by BER. PARP inhibitors prevent the separation of PARP from the broken DNA single strand, PARylation is inhibited and the BER pathway is impaired, SSBs will persist. A replication fork may encounter persistent SSBs during DNA replication, which causes the replication fork to collapse or the formation of DSBs. DSBs are usually repaired by HRR, when some key homologous recombination genes are damaged or dysregulated, HRD will occur. And then DNA cannot be required, or is repaired by alternative pathways that are highly error prone, which results in gross genomic instability and cell death. SSB: single-strand breaks; DSB: double-strands breaks: BER: base excision repair; HRR: homologous recombination repair; HRD: homologous recombination deficiency; NHEJ: non-homologous end joining: MMEJ: microhomology-mediated end joining.

PARPis have two main mechanisms of action: impairing SSB repair and capturing PARP (Figure 2). PARPis block the NAD + binding site on PARP, effectively inhibits the acylation of PARP, prevents the separation of PARP from the broken DNA single strand, and the base excision repair (BER) pathway is impaired, leading to the accumulation of unrepaired broken DNA single strands. The broken DNA single strands are not completely repaired, and the cell enters the S phase. At this time, PARPi captures PARP on the DNA chain, preventing replication bifurcation. In the absence of functional HRR, DSB will occur, which requires an effective repair mechanism to ensure genome stability (Pommier et al., 2016; D'Andrea, 2018; Murai et al., 2012). DSBs in HRD cells can only be treated by error-prone DNA repair mechanisms, such as non-homologous end joining (NHEJ) or microhomology-mediated end joining (MMEJ) (Ahmed et al., 2010). In the case of HRD, cells harboring *BRCA*m repair DSBs through NHEJ, resulting in harmful genomic instability. This mechanism is called synthetic lethality. Numerous studies have shown that PARPis benefits the treatment of newly diagnosed and relapsed *BRCA*m ovarian cancer. In addition to *BRCA* mutations, germline or somatic mutations of other HR genes, such as *ATM*, *CHEK2*, *BRIPD1*, *RAD51C*, and *PALB2*, may also become targets of PARPis acting on, making PARPis beneficial to a wider range of people (D'Andrea, 2018; Murai et al., 2012).

## Clinical Trials Results for PARP Inhibitors in Ovarian Cancer With Non-BRCA Mutations

**FIGURE 3:**
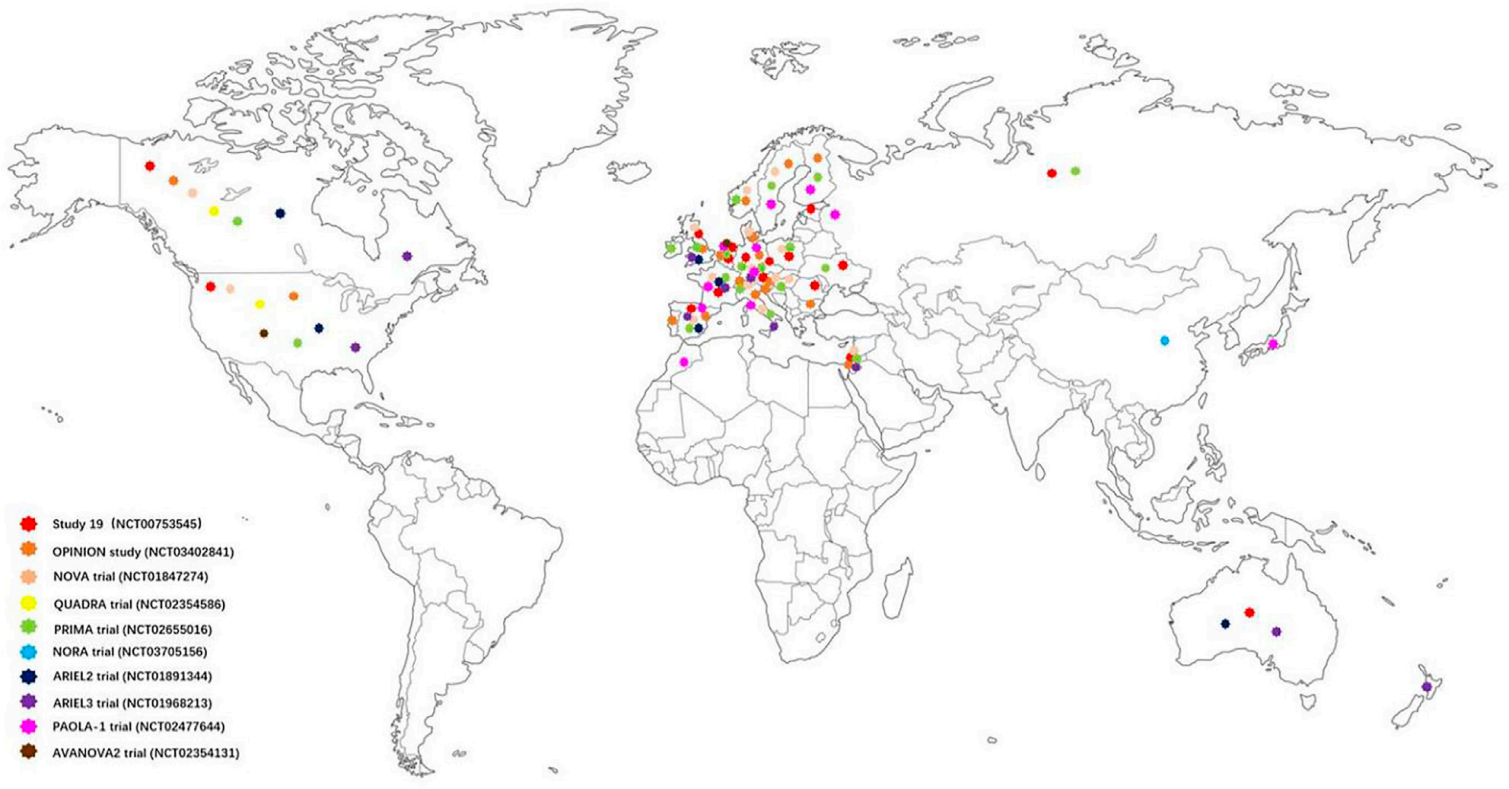
Geographical distribution map of the subjects in some selected key studies of PARP inhibitors in ovarian cancer with non-BRCA mutations. The subjects in study 19, OPINION study, NOVA trial, QUADRA trial, PRIMA trail, NORA trail, ARIEL2 trail, ARIEL3 trail, PAOLA-1 trail and AVANOVA2 trail are marked with dots of different colors. It can be seen that most of the subjects came from the United States, Canada, and some countries in Europe.

In recent years, many clinical trials have evaluated the efficacy of PARPis in the maintenance treatment of newly diagnosed and recurrent ovarian cancer after a complete response or partial response (CR/PR) to platinum-based chemotherapy. These trials concluded that PARPis significantly prolonged the PFS of ovarian cancer patients, and patients obtained a more satisfactory objective response rate (ORR). Although *BRCA*m ovarian cancer patients remain the main beneficiaries, HRD-positive ovarian cancer patients have also shown a surprising survival benefit, and HRD-negative ovarian cancer patients have also benefited from PARPis to some extent. The following review highlights the research status of the three PARPis (olaparib, niraparib and rucaparib) currently approved by the United States Food and Drug Administration (FDA) and the European Medicines Agency (EMA) in ovarian cancer with non-*BRCA* mutations. The indications approved by the FDA for the three PARPis are shown in Table 1, some published results for selected key studies of PARPis in ovarian cancer with non-*BRCA* mutations are shown in Table 2, and the geographical distribution of subjects in these key studies are shown in Figure 3.

**TABLE 1:** FDA approvals for PARP inhibitors in patients with ovarian, fallopian tube, and primary peritoneal cancers.

Drug	Indication	Year approved	Study ^(references)^
Olaparib	For the treatment of patients with deleterious or suspected deleterious gBRCAm advanced ovarian cancer having been treated with ≥3 prior lines of chemotherapy	2014	Study 42 ^Kaufman et al. (2015)^
	For the maintenance treatment of adult patients with recurrent epithelial ovarian, fallopian tube, or primary peritoneal cancer being in CR/PR to platinum-based chemotherapy	2017	SOLO-2^Pujade-Lauraine et al. (2017)^
			Study 19^Ledermann et al. (2012);^ ^Ledermann et al. (2016)^
	For the maintenance treatment of newly diagnosed BRCAm adult patients with advanced epithelial ovarian, fallopian tube, or primary peritoneal cancer being in CR/PR with first-line platinum-based chemotherapy	2018	SOLO-1^Moore et al. (2018a)^
Olaparib + bevacizumab	For the maintenance treatment of newly diagnosed HRD-positive adult patients with advanced epithelial ovarian, fallopian tube, or primary peritoneal cancer being in CR/PR with first-line platinum-based chemotherapy	2020	PAOLA-1^Ray-Coquard et al. (2019);^ ^Harter et al. (2020)^
Niraparib	For the maintenance treatment of adult patients with recurrent epithelial ovarian, fallopian tube, or primary peritoneal cancer being in CR/PR to platinum-based chemotherapy	2017	NOVA ^Mirza et al. (2016)^
	For the treatment of adult patients with recurrent epithelial ovarian, fallopian tube, or primary peritoneal cancer having been treated with ≥3 prior lines of chemotherapy and meet one of the following criteria: with BRCA mutation or HRD-positive and platinum sensitive	2019	QUADRA ^Moore et al. (2018b):^ ^Moore et al. (2019)^
	For the maintenance treatment of newly diagnosed adult patients with advanced epithelial ovarian, fallopian tube, or primary peritoneal cancer being in CR/PR with first-line platinum-based chemotherapy	2020	PRIMA ^González Martín et al. (2019)^
Rucaparib	For treatment of patients with deleterious g/sBRCAm associated advanced ovarian cancer having been treated with ≥3 chemotherapies	2016	ARIEL2 ^Swisher et al. (2017)^
			Study 10^Kristeleit et al. (2017)^
	For the maintenance treatment of recurrent epithelial ovarian, fallopian tube, or primary peritoneal cancer being in CR/PR to platinum-based chemotherapy	2018	ARIEL3 ^Coleman et al. (2017)^

*BRCA:* Breast-related cancer antigens, HRD: homologous recombination deficiency, CR: complete response, PR: partial response, *BRCA*m: *BRCA*-mutated, g*BRCA*m: Germline *BRCA*-mutated, s*BRCA*m: Somatic *BRCA*-mutated.

**TABLE 2:** Published results for selected key studies of PARP inhibitors in Ovarian Cancer with non-*BRCA* mutations.

Study ^(references)^	Phase	Study population	Treatment arm(s)	PFS(months)	ORR
Study 19 ^Ledermann et al. (2012),^ ^Ledermann et al. (2016)^	II	PSR HGSOC, irrespective of *BRCA* status (who had response to platinum-based chemotherapy)	Olaparib 400 mg Bid vs. placebo	Overall: 8.4 vs. 4.8 (HR 0.35; *p* < 0.001)	34%
				-*BRCA*m: 11.2 vs. 4.3 (HR 0.18; *p* < 0.0001)	
				-non-*BRCA*m: 7.4 vs. 5.5 (HR 0.54; *p* = 0.0075)	
OPINION ^Poveda et al. (2019),^ ^Poveda et al. (2020)^	III	PSR ovarian cancer patients without g*BRCA*m	Olaparib 300 mg Bid	Overall: was 9.2	NA
Light ^Cadoo et al. (2020)^	II	Patients with known *BRCA*m and HRD status (who previously received at least 1 previous line of platinum-based chemotherapy)	Olaparib 300 mg Bid	-g*BRCA*m: 11.0	-g*BRCA*m: 69%
				-sBRCAm:10.8	-s*BRCA*m: 64%
				-HRD-positive without BRCAm: 7.2	-HRD-positive without *BRCA*m: 29%
				-HRD-negative: 5.4	-HRD-negative:10%
NOVA ^Mirza et al. (2016),^	III	PSR ovarian cancer (who had response to the last platinum-based chemotherapy)	Niraparib 300 mg Qd vs. placebo	-g*BRCA*m: 21.0 vs. 5.5 (HR 0.27; *p* < 0.001)	NA
				-HRD-positive without g*BRCA*m: 12.9 vs. 3.8 (HR 0.38; *p* < 0.001)	
				-overall non-g*BRCA*m: 9.3 vs. 3.9 (HR 0.45; *p* < 0.001)	
QUADRA ^Moore et al. (2018b),^ ^Moore et al. (2019)^	II	Recurrent high-grade serous (grade 2 or 3) epithelial ovarian cancer patients (who received ≥3 prior chemotherapy regimens)	Niraparib 300 mg Qd	NA	28% (95%CI: 15.6–42.6, one-sided *p* = 0.00053)
PRIMA ^González Martín et al. (2019)^	III	Newly diagnosed advanced ovarian cancer (who had response to platinum-based chemotherapy)	Niraparib 300 mg Qd vs. placebo. starting dose of 200 mg Qd for patients with a baseline body weight <77 kg, a platelet count <150 × 10^3^/μL	-HRD-positive:21.9 vs. 10.4 (HR 0.43; *p* < 0.001)	NA
				-Overall: 13.8 vs. 8.2 (HR 0.62; *p* < 0.001)	
NORA ^Wu et al. (2021)^	III	Adult patients with platinum-sensitive recurrent ovarian cancer (who had response to their most recent platinum-containing chemotherapy)	Patients with a body weight <77 kg or a platelet count <150 × 10^3^/μL received Niraparib 200 mg Qd, and all other patients 300 mg Qd	Overall:18.3 vs. 5.4 (HR 0.32; 95%CI: 0.23–0.45; *p* < 0.0001)	Subgroup2
				Subgroup1	-g*BRCA*m: 50.0 vs. 28.6%
				-g*BRCA*m cohort (CR): NR vs. 5.49 (HR 0.12; *p* < 0.0001)	-*BRCA*wt: 25.8 vs. 7.1%
				-g*BRCA*m cohort (PR): 10.97 vs. 3.76 (HR 0.36; *p* = 0.0092)	
				-non-g*BRCA*m cohort (CR): 18.46 vs. 7.43 (HR 0.45; *p* = 0.0177)	
				-non-g*BRCA*m cohort (CR): 7.43 vs. 3.68 (HR 0.34; *p* < 0.0001)	
				Subgroup3	
				-relapsed 6–12 months after the penultimate chemotherapy: 11.2 vs. 3.7 (HR 0.31; *p* < 0.0001)	
				-relapsed ≥12 months after the penultimate chemotherapy: 18.4 vs. 5.5 (HR 0.33; *p* < 0.0001)	
ARIEL 2 part 1 ^Swisher et al. (2017)^	II	Patients with PSR high-grade (serous or endometroid) ovarian cancer (who previously treated with ≥1 lines of chemotherapy)	Rucaparib 600 mg Bid	-*BRCA*m: 12.8 (HR 0.27, *p* < 0.0001)	-*BRCA*m: 80%
				-*BRCA*wt/LOH high: 5.7 (HR 0.62; *p* = 0.011)	-*BRCA*wt/LOH high: 29%
				-*BRCA*wt/LOH low: 5.2	-*BRCA*wt/LOH low: 10%
ARIEL 3 ^Coleman et al. (2017),^ ^Ledermann et al. (2020)^	III	Platinum-sensitive recurrent disease (who had response to platinum-based chemotherapy)	Rucaparib 600 mg Bid vs. placebo	-*BRCA*m: 16.6 vs. 5.4 (HR 0.23; *p* < 0 0.0001)	NA
				-HRD-positive: 13.6 vs. 5.4 (HR 0.32; *p* < 0.0001)	
				-ITTP: 10.8 vs. 5.4 (HR 0.32; *p* < 0 0.0001)	

PFS: progression free survival, ORR: objective response rate, PSR: Platinum-sensitive relapsed, HGSOC: high grade serous ovarian cancer, OC: ovarian cancer, EOC: endometrioid ovarian cancer, *BRCA*: Breast-related cancer antigens, HRD: homologous recombination deficiency, *BRCA*m: *BRCA*, mutated, g*BRCA*m: Germline *BRCA*, mutated, *BRCA*wt: *BRCA*, wild type, LOH: loss of heterozygosity, CR: complete response, PR: partial response, ITTP: intention to treat population, NR: not reached, NA: not applicable, Bid: Twice a day, Qd: Once a day.

### Olaparib

Olaparib is the first PARPi approved for the treatment of ovarian cancer in clinical practice. Based on the results of Study42 (NCT01078662) (Kaufman et al., 2015), SOLO2 (NCT01874353) (Pujade-Lauraine et al., 2017) and SOLO1 (NCT01844986) (Moore K. et al., 2018), olaparib was approved for the treatment of newly diagnosed and recurrent *BRCA*m ovarian cancer. The efficacy of olaparib in non-*BRCA*m ovarian cancer has also been verified in some clinical trials. In a randomized, placebo-controlled, double-blind, phase 2 trial (Study 19) (NCT00753545) (Ledermann et al., 2012), 265 patients with platinum-sensitive recurrent (PSR) serous ovarian cancer who had received two or more courses of platinum-based chemotherapy and had responded to their latest regimen received olaparib (*n* = 136) or placebo (*n* = 129). The primary endpoint was PFS, and the PFS in the olaparib arm was significantly longer than that in the placebo arm [8.4 vs. 4.8 months; hazard ratio for progression or death (HR) 0.35; *p* < 0.001]. To answer the question of whether the efficacy of olaparib varies according to *BRCA* mutation status, the researchers conducted a retrospective preplanned analysis and showed a significant improvement in PFS among patients with *BRCA* mutations in the olaparib group compared with the placebo group (11.2 vs. 4.3 months; HR 0.18; *p* < 0.0001) (Ledermann et al., 2016). The PFS benefit was also significantly improved in patients without *BRCA* mutations (7.4 vs. 5.5 months; HR 0.54; *p* = 0.0075). Although the PFS benefit was less apparent in the non-*BRCA*m population than in the *BRCA*m population, this finding provided evidence that a proportion of patients with non-*BRCA*m can also benefit from PARPis. The OPINION phase ⅢB study (NCT03402841) (Poveda et al., 2019) evaluated the efficacy and safety of olaparib monotherapy in patients without germline *BRCA*m (g*BRCA*m) platinum-sensitive relapsed (PSR) ovarian cancer. Patients received olaparib until either progressive disease or intolerable toxicity. The primary endpoint was PFS, and the secondary endpoints included PFS with different HRD statuses and somatic *BRCA*m (s*BRCA*m) statuses. The interim analysis results showed that the primary endpoint median duration of progression-free survival (mPFS) was 9.2 months [95% confidence interval (CI): 7.6–10.9 months] (Poveda et al., 2020). The OPINION study reconfirmed the conclusion of Study19 that the benefit of olaparib was not limited to the *BRCA*m population based on practical data. The Light trial (NCT02983799) (Cadoo et al., 2020) was a phase II, open-label, multicenter study, which was the first prospective trial to examine olaparib in the treatment of patients with PSR ovarian cancer in subgroups of patients with known *BRCA*m and HRD status. *BRCA*m and HRD statuses were determined by the Myriad *BRCA* Analysis CDx test and My Choice HRD test. A total of 272 participants who had received at least 1 previous line of platinum-based chemotherapy were assigned to 4 study cohorts, which included those with g*BRCA*m (cohort 1; *n* = 75), s*BRCA*m (cohort 2; *n* = 26), HRD positivity without *BRCA*m (cohort 3; *n* = 68) and HRD negativity (cohort 4; *n* = 90). Patients received olaparib until either progressive disease or intolerable toxicity. The primary endpoint of the trial was ORR, whereas key secondary endpoints included disease control rate (DCR) and PFS. The results from the primary analysis indicated that olaparib induced a greater magnitude of benefits in patients who harbored *BRCA* mutations or were HRD positive compared with those who were HRD negative. The ORR with olaparib was 69% (95% CI: 58–80%) in cohort 1, 64% (95% CI: 43–82%) in cohort 2, 29% (95% CI: 19–42%) in cohort 3, and 10% (95% CI: 5–18%) in cohort 4. Additionally, the DCRs in cohorts 1 through 4 were 96% (95% CI: 89–99%), 100% (95% CI: 86–100%), 79% (95% CI: 68–88%), and 75% (95% CI: 65–84%), respectively. The median PFS in these subgroups was 11.0 months (95% CI: 8.3–12.2), 10.8 months (95% CI: 7.3–not evaluable), 7.2 months (95% CI: 5.3–7.6), and 5.4 months (95% CI: 3.7–5.6), respectively. Although the survival benefits of olaparib in the *BRCA*m population were greatest, HRD-positive (non-*BRCA*m) patients also received some survival benefits from olaparib. These clinical trial results provide further evidence to support the use of olaparib in non-*BRCA*m ovarian cancer. Pignata et al. reported the ORZORA trial (NCT02476968) at the 2021 annual meeting of the American Society of Gynecologic Oncology (SGO). This trial was an open-label, single-arm, multicenter study designed to evaluate the efficacy and safety of olaparib maintenance therapy in patients with PSR ovarian cancer with *BRCA*m or other gene mutations associated with non-*BRCA* HRR, and the primary endpoint was PFS. Patients with platinum-sensitive recurrent ovarian cancer who received ≥2 lines of platinum-containing chemotherapy achieved CR or PR and received 400 mg twice daily olaparib. The mPFS was 18.0 (95% CI: 14.3–22.1) months for the *BRCA*m cohort and 16.4 (95% CI: 10.9–19.3) months for the non-*BRCA* HRRm cohort, and maintenance therapy with olaparib showed a clinical benefit in PSR ovarian cancer patients with non-*BRCA* HRR mutations.

### Niraparib

Niraparib is a potent selective PARP1 and PARP2 inhibitor. The ENGOT-OV16/NOVA trial (NCT01847274) (Mirza et al., 2016), a randomized, double-blind, phase 3 trial, assessed the clinical benefits in patients with PSR ovarian cancer who exhibits a response to their last platinum-based chemotherapy. Patients were grouped by the presence or absence of g*BRCA*m and received niraparib or placebo. The primary endpoint was PFS. Patients in the niraparib group had a significantly longer mPFS than those in the placebo group in the g*BRCA*m cohort (21.0 vs. 5.5 months; HR 0.27; 95% CI: 0.17–0.41), the HRD-positive without g*BRCA*m cohort (12.9 vs. 3.8 months, HR 0.38, 95% CI, 0.24–0.59) and the overall non-g*BRCA*m cohort (9.3 vs. 3.9 months, HR 0.45, 95% CI: 0.34–0.61) (*p* < 0.001 for all three comparisons). Among PSR ovarian cancer patients, the mPFS of patients receiving niraparib was significantly longer than that of patients receiving placebo, regardless of the g*BRCA*m or HRD status. Niraparib was used as a maintenance therapy for PSR ovarian cancer based on the results of the ENGOT-OV16/NOVA trial. As the secondary endpoint of the NOVA trial, OS was affected not only by the studied drugs but also by subsequent treatment, cross-medication and other factors. After an average follow-up time of 5.6 years, the final analysis of the NOVA trial was performed. In the non-g*BRCA*m cohort, the median OS was 36.5 months in the placebo group and 31.1 months in the niraparib group (HR 1.10; 95% CI: 0.831–1.459). In the g*BRCA*m cohort, the median OS was 41.6 months in the placebo group and 43.6 months in the niraparib group (HR 0.93; 95% CI: 0.633–1.355). After adjusting for subsequent PARPi treatment using the inverse probability weighting method, the results of the analysis differed. In the non-g*BRCA*m cohort, the median OS was 35.9 months in the placebo group and 31.3 months in the niraparib group (HR 0.97; 95% CI: 0.74–1.26). In the g*BRCA*m cohort, the median OS was 34.1 months in the placebo group and 43.8 months in the niraparib group (HR 0.66, 95% CI: 0.44–0.99). This analysis concluded that there was no OS benefit in the non-g*BRCA*m cohort; however, in the g*BRCA*m cohort, niraparib maintenance treatment showed an advantage in improving OS (HR 0.66, median OS increased by 9.7 months) (Matulonis et al., 2021). The QUADRA trial (NCT02354586) (Moore et al., 2018; Moore et al., 2019) was a multicenter, open-label, single-arm, phase 2 study that evaluated the safety and activity of niraparib in relapsed HGSOC patients who had received ≥3 prior chemotherapy regimens. The primary objective was the proportion of HRD-positive patients achieving an overall response. Thirteen (27.5%) of 47 patients achieved an overall response according to RECIST (95% CI: 15.6–42.6; one-sided *p* = 0.00053). The QUADRA trial observed clinically relevant activity of niraparib among HRD-positive platinum-sensitive ovarian cancer patients, regardless of the status of *BRCA*m, supporting expansion of the treatment indication for PARPis to patients with HRD-positive ovarian cancer beyond those with *BRCA*m. Based on the results of the Quadra trial, the FDA approved niraparib for the treatment of *BRCA*m recurrent ovarian cancer or HRD-positive PSR ovarian cancer after treatment with three or more prior lines of chemotherapy. The PRIMA trial (NCT02655016) (González-Martín et al., 2019), a randomized, double-blind, phase 3 trial, enrolled patients with newly diagnosed advanced ovarian cancer to receive niraparib or placebo after a response to platinum-based chemotherapy. The primary endpoint was PFS. Among the HRD-positive patients, the mPFS was significantly longer in the niraparib group compared with the placebo group (21.9 vs. 10.4 months; HR 0.43; 95% CI: 0.31–0.59; *p* < 0.001). In the overall population, the corresponding PFS values were 13.8 and 8.2 months, respectively (HR 0.62; 95% CI: 0.50–0.76; *p* < 0.001). Among patients with newly diagnosed advanced ovarian cancer responding to platinum-based chemotherapy, patients who received niraparib had significantly longer PFS than patients who received placebo, regardless of the HRD status. Niraparib is the first PARPi approved for maintenance therapy in newly diagnosed advanced ovarian cancer regardless of *BRCA*m or HRD status. The NORA trial (NCT03705156) (Wu et al., 2021), a phase III, double-blind, placebo-controlled study, evaluated maintenance treatment with niraparib in PSR ovarian cancer patients who had responded to their most recent platinum-containing chemotherapy. Patients were stratified by *BRCA*m status, time to recurrence following penultimate chemotherapy, and response to most recent chemotherapy. The primary endpoint was PFS. In the intention-to-treat (ITT) population, mPFS was significantly longer for patients receiving niraparib versus placebo: 18.3 (95% CI: 10.9-not evaluable) versus 5.4 (95% CI: 3.7–5.7) months (HR 0.32; 95% CI: 0.23–0.45; *p* < 0.0001). A similar PFS benefit was observed in patients regardless of *BRCA*m status. PSR ovarian cancer patients with niraparib maintenance treatment had a statistically significant improvement in PFS regardless of *BRCA*m status. The latest three subgroup analyses of the NORA study was presented at the annual meeting of SGO in 2021. Of the 265 patients enrolled in the NORA study, 133 (50.2%) achieved CR after the last round of platinum-based chemotherapy (86 in the niraparib group and 47 in the placebo group), and 131 (49.4%) achieved PR (90 in the niraparib group and 41 in the placebo group). In the CR group of the g*BRCA*m cohort, mPFS was not reached (NR) (95% CI: 18.33-not evaluable) in patients receiving niraparib versus 5.49 (95% CI: 3.58–7.23) months for placebo (HR 0.12; 95%CI: 0.05–0.31; *p* < 0.0001). In the PR group, mPFS was10.97 months (95% CI: 7.39-not evaluable) in patients receiving niraparib versus 3.76 (95% CI: 1.87–7.36) months for placebo (HR 0.36; 95% CI: 0.16–0.81; *p* = 0.0092). In the CR group of the non-g*BRCA*m cohort, mPFS was 18.46 months (95% CI: 11.07-not evaluable) in patients receiving niraparib versus 7.43 (95% CI: 3.75-not evaluable) months for placebo (HR 0.45; 95% CI: 0.22–0.90; *p* = 0.0177). In the PR group, mPFS was 7.43 (95% CI: 5.55–11.01) in patients receiving niraparib versus 3.68 (95% CI: 1.87–5.49) months for placebo (HR 0.34; 95% CI: 0.20–0.58; *p* < 0.0001). Based on the above data, Yang et al. concluded that, compared with placebo, niraparib maintenance therapy significantly reduced the risk of disease progression in PSR ovarian cancer, regardless of CR or PR achieved by the last platinum-containing chemotherapy and regardless of g*BRCA*m status. Of the 265 patients in the NORA study, 64 (24.2%) had measurable residual lesions at baseline (43 in the niraparib group and 21 in the placebo group), and 21.9% (14/64) underwent secondary tumor reduction prior to chemotherapy. Yin et al. concluded at the meeting that in baseline platinum-sensitive relapsed ovarian cancer patients with measurable lesions, the niraparib group had a higher objective response rate (ORR) than the placebo group (ORR: 32.6 vs. 14.3%; OR = 2.7; 95% CI: 0.67–11.18). Among patients with g*BRCA*m or *BRCA* wild type (*BRCA*wt), compared with the placebo group, the niraparib group had a higher ORR (g*BRCA*m 50.0 vs. 28.6%, g*BRCA*wt 25.8 vs. 7.1%). Among 265 patients, 84 patients (31.7%) had recurrence 6–12 months after penultimate chemotherapy, and 181 patients (68.3%) experienced recurrence ≥12 months after chemotherapy. Among patients who relapsed 6–12 months after penultimate chemotherapy, the PFS of niraparib maintenance therapy versus placebo was 11.2 versus 3.7 months, respectively (HR = 0.31; 95% CI: 0.17–0.55, *p* < 0.0001). Among patients who relapsed ≥12 months after penultimate chemotherapy, the PFS of niraparib maintenance therapy versus placebo was 18.4 versus 5.5 months, respectively (HR = 0.33; 95% CI: 0.22–0.51; *p* < 0.0001). Huang et al. showed a significant PFS benefit in patients who relapsed 6–12 months and ≥12 months after penultimate chemotherapy, and the survival benefit was independent of g*BRCA*m status based on subgroup analysis.

### Rucaparib

ARIEL 2 Part 1 (NCT01891344) (Swisher et al., 2017), an international phase II trial, investigated the effectiveness of rucaparib in patients with PSR high-grade (serous or endometroid) ovarian cancer previously treated with ≥1 line of chemotherapy. A total of 192 patients were stratified into three HRD subgroups: *BRCA*m (*n* = 40), *BRCA*wt with high loss of heterozygosity (LOH) (*n* = 82), and *BRCA*wt with low LOH (*n* = 70). Compared to the *BRCA*wt/LOH low subgroup (5.2 months), the mPFS was significantly longer in the *BRCA*m subgroup (12.8 months; HR 0.27, *p* < 0.0001) and the *BRCA*wt/LOH high subgroup (5.7 months; HR 0.62, *p* = 0.011). The ORR was higher in the *BRCA*1/2-m (80%) and *BRCA*wt/LOH high subgroup (29%) compared with the *BRCA*wt/LOH low subgroup (10%). This study identified LOH as a predictive molecular biomarker for measuring HRD. ARIEL3 (NCT01968213) (Coleman et al., 2017; Ledermann et al., 2020) was developed as a phase III, double-blinded, randomized trial to assess the efficacy of rucaparib compared to placebo in patients with PSR ovarian cancer who also achieved CR/PR to their last line of platinum chemotherapy. The primary endpoint was PFS, which was tested for three nested cohorts: 1) g/s*BRCA*m patients; 2) patients with HRD (*BRCA*m or *BRCA*wt and high LOH); and 3) intention to treat (ITT) population. In the *BRCA*m group, the mPFS was significantly longer in the rucaparib group compared to the placebo group (16.6 vs. 5.4 months; HR 0.23; 95% CI: 0.16–0.34); *p* < 0.0001). In HRD-positive patients, the mPFS of patients received rucaparib versus placebo (13.6 vs. 5.4 months HR 0.32; 95% CI: 0.24–0.42; *p* < 0.0001), and in the ITT population, the mPFS of patients received rucaparib versus placebo (10.8 vs. 5.4 months; HR 0.36; 95% CI: 0.30–0.45; *p* < 0.0001). The results from ARIEL3 were consistent with those of the ENGOT-OV16/NOVA trial, indicating efficacy in maintenance treatment for PSR ovarian cancer regardless of *BRCA*m status. Based on these data, the FDA expanded rucaparib indications to the maintenance treatment of recurrent epithelial ovarian cancers achieving CR or PR to platinum-based chemotherapy.

## Overcoming Resistance to PARP Inhibitors

Despite the significant survival benefits of PARPi in the maintenance treatment of ovarian cancer, the problem of resistance to PARPi is emerging. Currently, it is believed that the two main mechanisms of PARPi resistance in tumor cells include the recovery of HR and the protection of replication forks (Weigelt et al., 2017; Francica and Rottenberg, 2018). Tumor cells can reverse HR gene mutations; thus, tumor cells become proficient in HR and resistant to PARPi. Reversions of key HR genes, including *BRCA*1, *BRCA*2, *RAD51C*, and *RAD51D*, were observed in cell line models (Sakai et al., 2008; Swisher et al., 2008; Sakai et al., 2009; Bitler et al., 2017; Kondrashova et al., 2017). Tumor cells can also restore HR by inhibiting the HR antagonistic pathway NHEJ. Given that HRD cells cannot repair DSBs through HR, NHEJ increases in these cells, and NHEJ deficiency leads to resistance to PARPis (Kondrashova et al., 2017). When the key proteins protecting the replication fork are lost, the unstable replication fork leads to the production of DSBs, which provide a target for chemotherapy, such as platinum and PARPi, and then the tumor cells stabilize replicate forks activate.

At present, studies focus on the recovery of PARPi sensitivity through combination therapy, including combining PARPis with antiangiogenic drugs, immunotherapy or other biological agents. Table 3 shows published results for selected key studies of combination therapy to overcome resistance to PARP inhibitors.

**TABLE 3:** Published results for selected key studies of combination therapy to overcome resistance to PARP inhibitors.

Study ^(references)^	Phase	Study population	Treatment arm (s)	PFS (months)	ORR
PAOLA-1 ^Ray-Coquard et al. (2019),^ ^Harter et al. (2020)^	III	Newly diagnosed, advanced, high-grade ovarian cancer (who had response to first-line platinum-taxane chemotherapy plus bevacizumab) regardless of surgical outcome or *BRCA*m status)	Olaparib 300 mg Bid + bevacizumab (15 mg per kilogram of body weight every 3 weeks) vs. placebo + bevacizumab	Overall: 22.1 vs. 16.6 (HR 0.59; *p* < 0.001)	NA
				- HRD-positive without BRCAm: 37.2 vs. 17.7 (HR 0.33)	
				-HRD-negative: HR 1.00	
AVANOVA2 ^Mirza et al. (2019),^ ^Mirza et al. (2020)^	II	Patients with PS EOC regardless of HRD status	Niraparib 300 mg Qd + bevacizumab (15 mg per kilogram of body weight every 3 weeks) vs. niraparib 300 mg Qd	Overall: 11.9 vs. 5.5 (HR 0.35; *p* < 0.0001)	NA

PFS: progression free survival, ORR: objective response rate, EOC: endometrioid ovarian cancer, *BRCA*: Breast-related cancer antigens, HRD: homologous recombination deficiency, *BRCA*m: *BRCA*, mutated, NA: not applicable, Bid: Twice a day, Qd: Once a day.

### PARPis and Antiangiogenic Agents

Antiangiogenic therapy induces cell hypoxia, which leads to downregulation of HR repair genes (*BRCA*1, *BRCA*2, and *RAD*51), increasing tumor sensitivity to PARPis (Bindra et al., 2005). The therapeutic principle of inducing HRD by combining PARPi with drugs that can downregulate HR (such as tyrosine kinase inhibitor of vascular endothelial growth factor receptor) is called “situational” synthetic lethal therapy (Papa et al., 2016). Cediranib is an oral tyrosine kinase inhibitor of vascular endothelial growth factor receptor (VEGFR). A randomized phase 2 study assessed the efficacy of combination cediranib and olaparib versus olaparib monotherapy in women with PSR ovarian cancer (Liu et al., 2014). The median PFS in patients who received cediranib plus olaparib was significantly longer than the mPFS in patients who received olaparib alone (17.7 vs. 9 months; HR 0.42; *p* = 0.005). A post-hoc exploratory analysis showed an increased therapeutic benefit of cediranib plus olaparib vs olaparib alone in the subgroup of patients with *BRCA*wt or unknown *BRCA* status with an improved mPFS from 5.7 to 16.5 months (HR = 0.32, *p* = 0.008) and an improved ORR from 32 to 76% (*p* = 0.006). Among g*BRCA*m patients, there was a lesser trend towards increased therapeutic benefits for the combination arm with a lower gain of PFS (from 16.5 to 19.4 months) and ORR (benefit from 63 to 84%). When PARPis are combined with antiangiogenic agents to overcome the drug resistance of ovarian tumor cells, the benefit may be more significant in the non-*BRCA*m population. PAOLA-1/ENGOT-ov25 (NCT02477644) (Ray-Coquard et al., 2019; Harter et al., 2020) is a randomized, double-blind, international phase 3 trial that enrolled patients who were newly diagnosed with high-grade ovarian cancer and had a response after first-line platinum-taxane chemotherapy plus bevacizumab regardless of surgical outcome or *BRCA*m status. This study showed that by adding olaparib to first-line bevacizumab maintenance therapy, PFS was significantly improved. The mPFS was 22.1 months in patients treated with olaparib plus bevacizumab, and the mPFS in patients treated with placebo plus bevacizumab was 16.6 months (HR 0.59; 95% CI: 0.49–0.72; *p* < 0.001). Prespecified subgroup analyses revealed that the group of patients with HRD-positive tumors (including those with *BRCA*m) derived the greatest benefit. PFS in patients who received olaparib plus bevacizumab was longer compared to patients who received placebo (37.2 vs. 17.7 months; HR 0.33). In patients with HRD positivity and without *BRCA*m, the addition of olaparib to bevacizumab maintenance therapy also resulted in a significant extension in PFS. However, HRD-negative patients did not derive any clinically significant benefit (HR 1.00; 95% CI: 0.75–1.35). Of note, no patients received olaparib monotherapy, and comparisons of the benefits of olaparib monotherapy and the combination therapy of olaparib and bevacizumab cannot be made. Based on these results, FDA approval was gained for olaparib in combination with bevacizumab for first-line maintenance therapy for newly diagnosed advanced ovarian cancer patients who were HRD positive. ENGOT-OV24-NSGO/AVANOVA2 (NCT02354131) (Mirza et al., 2019; Mirza et al., 2020) is a two-arm, open-label phase II, randomized study of niraparib versus the niraparib/bevacizumab combination in patients with PS EOC. The primary endpoint is PFS. The available data showed significant improvement in mPFS in patients who received niraparib plus bevacizumab compared with niraparib alone, regardless of HRD status (11.9 vs. 5.5 months; HR 0.35; 95% CI 0.27–0.57; *p* < 0.0001). Two phase III trials are currently ongoing to validate this combination in different settings. The GY004 trial (NCT02446600) intended to explore and compare the benefits of three therapeutic regimens (olaparib monotherapy, the combination of olaparib and cediranib, standard platinum-based chemotherapy) in patients with PSR ovarian cancer. The ICON9 trial (NCT03278717) is examining maintenance therapy with a combination of cediranib and olaparib or olaparib alone after platinum-based chemotherapy in patients with PSR high-grade ovarian cancer. More detailed clinical data of the two trials are expected.

### PARPis and Immune Checkpoint Inhibitors

The efficacy of PARPi in combination with immunotherapy is also being studied in clinical trials. DNA damage activates the interferon gene stimulating factor (STING) pathway, which plays a key role in innate immunity by inducing the production of type I interferon and proinflammatory cytokines (Barber, 2015). PARPi enhances the response of HRD-positive OC to immunotherapy by generating a greater immune burden and amplifying the expression of neoantigens. The main immune checkpoint inhibitors currently are monoclonal antibodies against programmed death protein 1 (PD-1) or programmed death ligand 1 (PD-L1). The combination of PARPi and immune checkpoint inhibitors is promising in patients with HDR-positive EOC (Mittica et al., 2016). *BRCA*m and non-*BRCA*m HRD ovarian tumors show a higher neoantigen load than HR-proficient cancers (Strickland et al., 2016), thereby enhancing the recruitment of tumor-infiltrating lymphocytes (TILs). These tumors typically present with elevated CD3^+^ and CD8^+^ TILs and increased PD-1 and PD-L1 expression and are more sensitive to PD-1/PD-L1 inhibitors. Thus, these tumors represent a subset of tumors suitable for combination therapy with immune checkpoint inhibitors and PARPis. The phase I/II TOPACIO trial (NCT02657889) (Konstantinopoulos et al., 2018) showed that nilaparib in combination with pembrolizumab is a promising option for the treatment of platinum-resistant OC. Preliminary efficacy data showed that 13 of 49 patients responded to combination therapy with similar adverse events as those in the previous monotherapy study. Interestingly, 77% of patients were *BRCA* wild type and 52% were HRD negative with objective response rates of 24 and 27%, respectively, suggesting that the combination therapy was active in the HRD-negative population. ATHENA trial (NCT03522246) is a study evaluating rucaparib and nivolumab (anti-*PD-1*) maintenance treatment following front-line platinum-based chemotherapy. It is a phase III, randomized, double-blind, dual placebo-controlled, four-arm study stratified based on platinum-based therapy, germline/somatic *BRCA* status, loss of heterozygosity, and timing of tumor reduction surgery. The primary endpoint is PFS. This trial has completed enrollment, and the data are being analyzed. In addition, the OPAL study explored the efficacy of niraparib + bevacizumab + TSR042 (a *PD-1* inhibitor) in ovarian cancer patients with PSR.

### PARPis and Other Agents

The combination of PARPis and molecular targeted drugs that inhibit HRR has also become a research direction for overcoming PARPi resistance (Wilson et al., 2016). Studies have shown that phosphoinositide 3-kinase (PI3K) inhibitors significantly reduce the expression of HR-related genes, leading to acquired HRD, which is the basis of the antitumor effect of PARPis (Ibrahim et al., 2012; Juvekar et al., 2012). A phase I evaluation of PI3K inhibitors for ovarian cancer and triple-negative breast cancer has been completed (Konstantinopoulos et al., 2015). The combination of olaparib and BMK120 in 46 patients with advanced ovarian cancer had an ORR of 29%.

Topotecan, a topoisomerase inhibitor, induces replication fork instability and promotes DNA damage. Topotecan combined with PARPi may have an anti-drug resistance effect, and this treatment strategy has been actively explored in clinical studies.

## HRD Status Detection

*BRCA* mutation testing is a routine test for ovarian cancer. Given the good performance of PARPi treatment in HRD-positive ovarian cancer patients, the use of PARPis is no longer limited to *BRCA*m ovarian cancer patients, and it is necessary to conduct HRD status detection in a large population of non-*BRCA*m ovarian cancer patients to identify ideal patient populations that will benefit from PARPis. The molecular and genomic changes that lead to HRD phenotypes are complex, and the current challenge is to establish reliable and unified HRD status detection methods in the context of non-*BRCA*m.

“Genome scar” is a pattern of genome mutation, insertion/deletion, and rearrangement, which reflects the accumulation of different processes of DNA damage and repair over time (Lord and Ashworth, 2012). “Genomic scar” represents the historical record of DNA damage exposure and tumor cells trying to reduce DNA damage through different DNA repair processes. Therefore, the genomic scar usually reflects specific DNA repair deficiency of tumor cells. The genomic scars caused by low fidelity repair of HRD are the basis of HRD status detection. Two major commercial assays were developed for assessing HRD status via genomic scarring patterns. MyChoice® CDx, the first commercially available HRD assay, was designed to determine HRD status through detection and classification of BRCA1/2 (sequencing and large rearrangement) variants and assessment of genomic instability combining three parameters: loss of heterozygosity (LOH), telomeric allelic imbalance, and large-scale state transitions. By combining these three independent measures of HRD, prognostic power is increased compared with any of the individual components. HRD positivity was defined when the HRD score cutoff was ≥42 (Telli et al., 2016). Foundation Focus® CDx tests tumor DNA to detect mutations in BRCA1/2 genes and the percentage of the genome affected by LOH. HRD positivity is noted if the LOH score is ≥16% (Watkins et al., 2014).

Since these HRD status detection methods vary in the precise measurement of genomic characteristics, they may not include the same group of patients. HRD status detection methods based on genomic scar evidence have been gradually applied to clinical trials of ovarian cancer to determine the subgroup of patients with HRD and evaluate the relationship between HRD and PARPi response. However, genomic HRD analysis is not a direct detection of HRD function. The HR function of tumor cells changes dynamically during treatment, while the genomic scar is permanent, HRD status detection may not represent the current HRD state of cancer cells. Emerging methods to detect HRD, including genomic and functional assays, may overcome this challenge. Currently, new assays are undergoing clinical validation, including 1) somatic mutations in homologous recombination genes, 2) “genomic scar” assays using array-based comparative genomic hybridization (aCGH), single nucleotide polymorphism (SNP) analysis or mutational signatures derived from next-generation sequencing, 3) transcriptional profiles of HRD, and 4) phenotypic or functional assays of protein expression and localization (Hoppe et al., 2018).

## Toxicities of the Three PARPis

Pharmacokinetics and pharmacodynamics of niraparib have been shown to be metabolised in the liver by carboxylesterase-catalysed amide hydrolysis, whereas rucaparib and olaparib are primarily metabolised by the cytochrome P450 enzymatic pathway (CYP) (Zhang et al., 2017).

We mainly reviewed three phase 3 maintenance trials: the SOLO 2 study (Pujade-Lauraine et al., 2017), the ENGOT-OV16/NOVA trial (Mirza et al., 2016), and the ARIEL3 trial (Coleman et al., 2017). Anaemia is the most common haematological toxicity among the three PARPis, grade 3 and 4 adverse events were slightly higher for niraparib [93 (25%) of 367 patients], followed by rucaparib [70 (19%) of 372 patients] and olaparib [38 (19%) of 195 patients]. Neutropenia was the third most common haematological toxicity observed, grade 3 and 4 adverse events were higher with niraparib [72 (20%) of 367 patients] compared with rucaparib [25 (7%) of 372 patients] and olaparib [10 (5%) of195 patients]. Thrombocytopenia of any grade is also more pronounced with niraparib. In general, all patients starting a PARPi or those who undergo a dose modification should have a complete blood count with differential monthly to monitor haematological toxicity. Gastrointestinal adverse events are common for the three PARPis, with nausea being the most prevalent. Only 3–4% of patients had grade 3 or 4 nausea. Another common adverse event that occurs with PARP inhibitors is an increase in creatinine concentrations. Rucaparib use in the ARIEL3 trial resulted in an elevation of creatinine (any grade) in 15% of patients versus 2% in the placebo group. In the SOLO 2 trial, 21 (11%) of 195 patients treated with olaparib had grades 1 or 2 elevations in creatinine (no grades 3 and 4) compared with 1% in the placebo group. Notably, niraparib was not associated with elevated serum creatinine. Fatigue is nearly universal toxicity for all PARP inhibitors. Non-pharmacological treatments, such as exercise, massage therapy, and cognitive behavioural therapy, can be effective in reducing symptoms. Gong et al., 2020 used network meta-analysis to, directly and indirectly, compare the toxicities of the three PARPis. The primary outcomes regarding toxicity reported in all studies were ORs for total grade 3–4 adverse events. The results showed that all assessed PARPi regimens significantly increased the number of grade 3–4 adverse events in ovarian cancer patients responsive to front-line platinum (OR: 1.94, 95%CI: 1.54–2.47 for bevacizumab + olaparib; OR: 2.18, 95%CI 1.42–3.50 for olaparib) or PSR patients (OR: 1.97, 95%CI 1.71–2.27 for olaparib; OR: 2.16, 95%CI 1.97–2.37 for rucaparib; and OR: 2.04, 95%CI 1.84–2.26 for niraparib) compared with placebo. Gong et al. observed no statistically significant differences in the ORs for total grade 3–4 adverse events.

## Future Directions

HRD positivity is a good indicator to screen people who can benefit from PARPis. However, due to the change in HR status during treatment, the negative prediction effect of the HRD test is poor. Repeated multiperiod detection of HRD status may help to avoid false negatives to improve the screening rate of PARPi beneficiaries and guide the judgment of the recovery status of HR to solve the problem of drug resistance through drug combinations in a timely manner and help patients obtain a better prognosis. However, such a strategy may be limited by the high cost of testing and the difficulty of determining when testing should occur. In addition, the identification of potential biomarkers other than HRD status to identify HRD-negative patients who will benefit from PARPis requires further research.

PARPis are currently used for maintenance treatment in patients with newly diagnosed ovarian cancer and for maintenance treatment in recurrent ovarian cancer. Many clinical trials exclude patients who have previously used PARPi, so data on the efficacy or safety in patients retreated with PARPis after previous PARPi treatment are limited. None of the approved drugs included indications for reuse of PARPi. A small retrospective study showed that previous use of PARPi 1 did not lead to drug resistance to subsequent use of PARPi 2, and repeated use of PARPi in the case of recurrence seemed to be a safe option (Essel et al., 2021). Currently, the prospective randomized controlled trial OREO/ENGOT OV-38 (the retreatment of olaparib in platinum-sensitive relapsed ovarian cancer) is investigating the issue of PARPi followed by PARPi monotherapy. The study began in 2017 with initial data expected in 2021.

Further research is needed to determine whether PARPi can be reused in the treatment of ovarian cancer, how the efficacy and toxicity of repeated use of PARPi will change, and the optimal time to use PARPi during the treatment cycle. The most feasible method to assess this problem is to conduct larger phase III trials to compare the efficacy, side effects, and tolerability of different PARPis Pellegrino et al., 2019.

## Conclusion

The existing trial results show that although *BRCA*m ovarian cancer patients still benefit the most from PARPi, the non-*BRCA*m population can indeed gain survival benefits from PARPi. The indications for PARPi will continue to expand, and the application of PARPi in the treatment of non-*BRCA*m ovarian cancer is expected. Based on the different biomarker statuses of ovarian cancer patients, the degree of benefits from PARPis is different, so it is necessary to explore a reliable and unified biomarker detection method to screen patients and select the appropriate PRAPi. How to accurately screen drug users, select appropriate PARPis, rationally combine drugs to overcome acquired drug resistance, and select appropriate medication time to maximize the treatment effect of PARPis is a research direction with high demand and great promise for further exploration.
